# Use of the Symani® microscopic surgical robot in hand surgery and operating room setup

**DOI:** 10.1007/s11701-025-02629-2

**Published:** 2025-08-27

**Authors:** Alessandro Piperno, Enrico Palombo, Alessia Pagnotta

**Affiliations:** 1 Hand and Microsurgery Unit, Jewish Hospital of Rome, Rome, Italy; 2 Department of Anatomical, Histological, Forensic Medicine and Orthopaedic Sciences, Sapienza University of Rome, Rome, Italy

**Keywords:** Robotic surgery, Symani®, Hand microsurgery, Reconstructive microsurgery, Robotic surgery setting

## Abstract

Robotic surgery has introduced significant innovations in hand microsurgery, enabling complex procedures such as nerve sutures and arterial and venous anastomoses to be performed with greater precision and control. The Symani® Surgical System represents a promising advancement over conventional techniques by offering enhanced dexterity, tremor elimination, and motion scaling to improve microsurgical outcomes. While most studies in the literature focus on clinical comparisons between traditional and robotic approaches, our work aims to explore the practical aspects of integrating the Symani® into hand surgery. Specifically, we describe the structured training pathway for both surgeons and nursing staff, along with a detailed setup of the operating room, including optimal team positioning and coordination. Our early experience suggests that, following a dedicated learning curve, the Symani® system can be successfully implemented in microsurgical clinical practice. Proper team training and operating room organization are essential to maximize the system’s benefits in terms of safety, efficiency, and surgical precision.

**Supplementary Information:**

The online version contains supplementary material available at 10.1007/s11701-025-02629-2.

## Introduction

Technological advancements in the healthcare field have led to increasingly sophisticated tools. In microsurgery, robotic systems such as Symani® help overcome technical limitations. Designed for both microsurgery and supermicrosurgery, Symani® offers several key advantages:

1. Elimination of essential tremor
2. Improved quality in vascular anastomoses and nerve sutures
3. Increased precision through motion scaling, which allows for:
  3.1. More controlled movements
  3.2. Greater surgical accuracy
4. Reduction of surgeon fatigue
5. Lower risk of intraoperative complications

The system includes a cart with macro-positioners and micromanipulators that support NanoWrist instruments in two sizes. Correct positioning of the robot and microscope is crucial for workspace optimization and procedural safety. The surgeon controls the robotic arms from a dedicated chair using foot pedals and ergonomic joysticks.

Motion scaling translates the surgeon’s hand motions into more controlled and refined actions, improving surgical accuracy. The aim of this study is to evaluate the feasibility and early integration of the Symani® Surgical System in hand microsurgery, with a focus on team training, operating room setup, and multi-user usability. While existing literature primarily compares outcomes between robot-assisted and conventional microsurgical techniques [1, 2], our work provides a detailed overview of the operating room setup, which is essential for preventing intraoperative challenges related to suboptimal positioning of the team, robot, and microscope. Additionally, it highlights the training process necessary for the entire surgical team to effectively integrate the Symani® system.

## Methods

This study is structured as a narrative implementation report based on our initial clinical experience with the Symani® Surgical System in hand microsurgery. The System Usability Scale (SUS) questionnaire was used to assess the overall usability of the device. It was completed by the lead surgeon, the assistant surgeon, and two operating room nurses involved in the procedures. At our center, the Symani® system was used in a hybrid configuration with the Zeiss OPMI Vario S88 microscope. A total of nine procedures were performed on eight patients, following dedicated training undertaken by the entire surgical team to learn how to operate the system:

- Surgeon training: 80 h, including 10 vascular anastomoses
- Nursing staff training: 15 h, covering component assembly, sterile draping of components, and troubleshooting
- Joint team training: Approximately 10 h between the surgeon and nurses on real-case simulations to replicate the setup and workflow

**Fig. 1:**
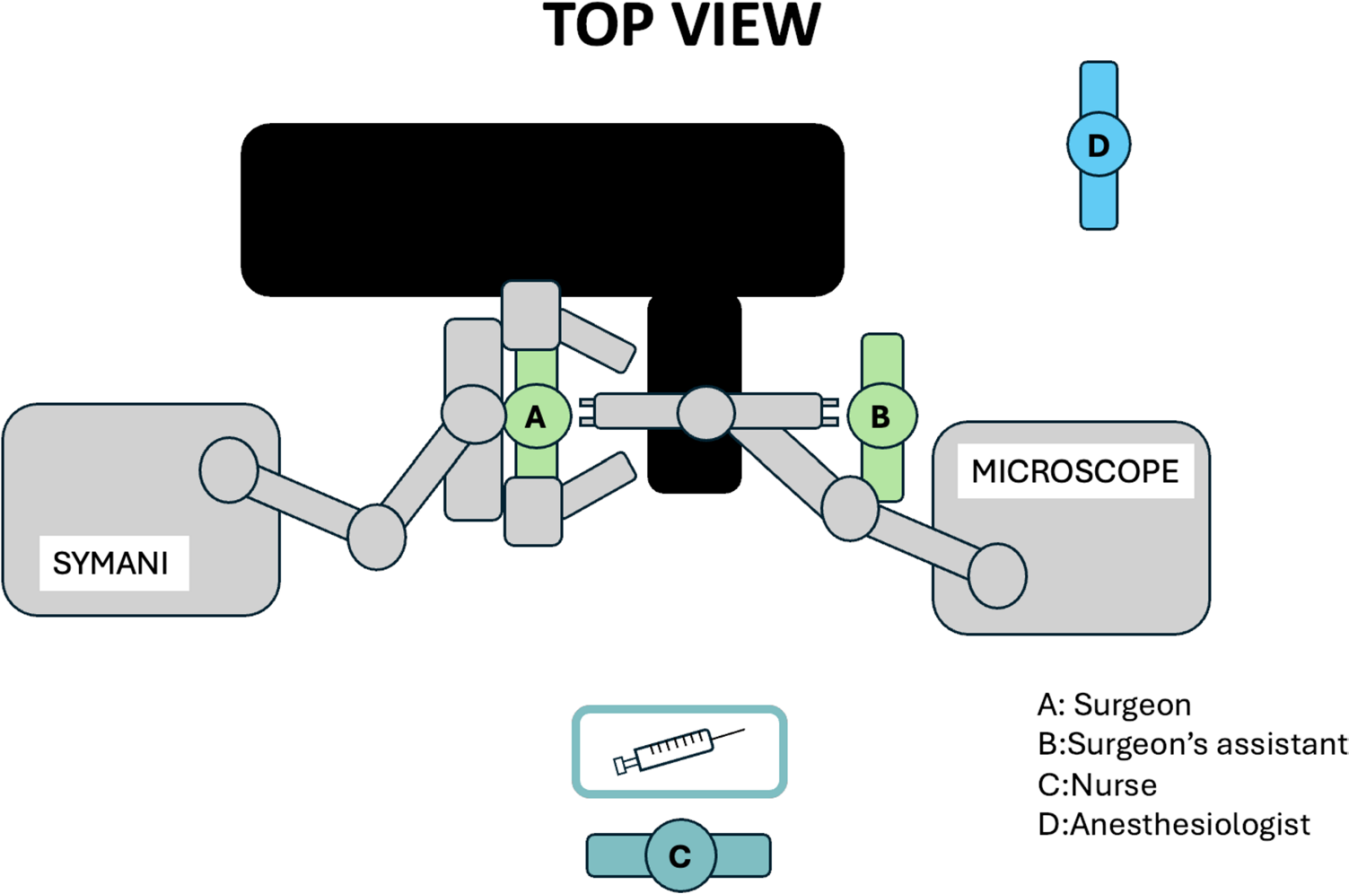
Schematic representation of the operating room setup. The patient lies in supine position with the upper limb abducted at 90°. The lead surgeon is seated with the robot positioned behind him (southeast). The microscope is located to the northeast of the lead surgeon. The assistant surgeon faces the lead surgeon, standing next to the microscope. The nurse is positioned between the two surgeons

In our experience with hand and upper limb surgery, the positioning of the robot and surgical team was organized as follows (Fig. 1):

- Patient in supine position
- Upper limb abducted at 90°
- Lead surgeon seated with the robot behind (southeast relative to the lead surgeon)
- Microscope placed northeast of the lead surgeon
- Assistant surgeon located across from the lead surgeon, next to the microscope
- Nurse positioned between the two surgeons

We conducted multiple hours of high-fidelity simulations and volunteers acted as a mock patient, allowing us to test and refine the appropriate operating room setting under realistic conditions. This iterative process underwent several adaptations and led to the creation of a standardized nursing checklist for device setup. The steps are summarized below:

1. Connect cables
2. Start up the system
3. Move the macro-positioner from the stowed position
4. Center the robotic arms and electric motors (no instruments attached)
5. Drape the robot and Symani® chair
6. Check NanoWrist instruments (Tip cap closed and correctly oriented; backend cap removed and pin inspected)
7. Slide instruments into the instrument connectors
8. Engage instruments with tip cap on
9. Position the microscope (northeast of the lead surgeon)
10. Position the Symani® robot (southeast of the lead surgeon), place the NanoWrist instruments near the target anatomy, and hand them over to the surgeon.
11. Remove tip caps and verify tips are fully closed
12. Lock the wheels
13. Begin teleoperation
14. After use, remove the micromanipulators from the operating field
15. Disengage instruments
16. Remove instruments
17. Remove drapes
18. Return the system to the stowed position
19. Shut down the system
20. Disconnect cables

Sutures were performed entirely with the robot or using a hybrid approach: starting with the first two stitches using traditional technique, followed by robotic suturing to increase confidence and optimize time.

### Surgical checklist for hand procedures

1. Isolation of vascular pedicles and nerves
2. Microscope positioning
3. Dissection of pedicles
4. Symani® positioning
  4.1. Sit on the draped Symani® chair and adjust height and elbows positions
  4.2. Position the Symani® southwest of the surgeon
  4.3. Place the Symani® arms close to the surgical field and position the head between them
  4.4. Align the microscope in front of the surgeon’s head
  4.5. Place the Symani® pedals under the right foot
  4.6. Position the microscope foot pedal (if present) under the left foot
  4.7. Take the Symani® pliers
  4.8. Make the “connection” holding the blue pedal and rotating the pliers until the continuing sound stops
5. Venous anastomosis (if dorsal)
6. Arterial and venous anastomosis/nerve sutures
7. Check anastomosis patency
8. Remove clamps

## Results

Usability was assessed using the System Usability Scale (SUS), a well-established tool for measuring perceived usability [3]. Two surgeons and two nurses completed the questionnaire (see supplementary material), but due to the limited number of respondents (*n* = 4), statistical analysis was not performed, as the sample size does not allow for meaningful interpretation. Instead, individual scores and averages are reported to reflect usability perceptions. Surgeons reported SUS scores of 77.5 and 72.5 (average 75.0, Category B – acceptable), while nurses scored 65.0 and 67.5 (average 66.25, Category C – sufficient but improvable). The higher scores from surgeons may reflect greater familiarity with the system. Despite this, both groups rated usability above the acceptability threshold, supporting its clinical suitability and highlighting areas for improvement, especially for nursing staff. Based on our experience, setting up the Symani® robotic system takes approximately two minutes to transition the robot from OFF to ON, with the software ready and the console connected. An additional 10–15 min on average are required to drape the robot and console and to mount the Nanowrist instruments onto the robot.

## Discussion

This study’s approach was essential for an effective introduction of the new robotic system. Shared training familiarized the entire team with the device, its features, and potential intraoperative issues. The positioning of the microscope, the robot, and the staff in the operating room plays a crucial role in ensuring the smooth surgical process and procedural safety. Positioning errors can compromise intervention efficiency, prolong operative time, and increase complications. The development of surgical and nursing checklists has significantly contributed to standardizing procedures, reducing variability, and improving reproducibility. These findings align with existing literature emphasizing team preparation and standardized workflows for the safe adoption of new surgical technologies. Previous studies highlight that the implementation of advanced robotics requires not only technical skills from the surgeon but also effective integration with the entire surgical team [4]. The assisting surgeon also plays an important role by managing sutures, maintaining a blood-free field, and repositioning the needle in the center. In hand surgery, current indications for microsurgery robots include vascular, nerve, and lymphovenous anastomoses in traumatic and oncologic microvascular reconstructive procedures [5]. With the ongoing development of new robotic tools for dissection, cauterization, and osteotomy, future applications may include delicate soft tissue dissection and vascularized bone grafting for conditions, such as non-union and bone necrosis [6,7].

It should be noted that the robot lacks a visual output system and relies on microscope or 3D exoscope visualization. Additionally, the robot does not provide haptic feedback; however, literature suggests this is not critical, as tactile sensation is minimal and visual control primarily guides precision for 9–0 sutures or finer [8].

Limitations of this study include a small, single-center sample of clinical cases, absence of long-term follow-up and outcome data, and a descriptive design without comparative statistics. These could limit the generalizability of our findings and conclusions about clinical effectiveness. Future research should aim to validate the integration of the Symani® system through multicenter studies, a formal evaluation of the learning curve, and the development of standardized training protocols for surgical proficiency. Additionally, future studies should assess patient-centered outcomes, such as functional recovery and quality of life. Our contribution focuses on an often-overlooked aspect: team training and operating room setup. While previous studies have focused more on clinical outcomes, few have examined the organizational elements affecting robotic surgery safety and quality. Surgical team feedback, though limited, suggests that early attention to training and setup can significantly reduce learning curves and intraoperative stress.

## Conclusions

Integrating the Symani® Surgical System into hand microsurgery requires structured team training and meticulous operating room organization. Our experience demonstrates that simulation-based sessions and coordinated involvement of both surgeons and nursing staff are essential for safe, efficient implementation. Our training protocol promoted familiarity with robotic instruments and established a shared workflow that enhanced communication and efficiency. After the learning curve, the system can be successfully incorporated into clinical practice, enabling more precise anastomoses through motion scaling, tremor reduction, and decreased surgeon fatigue. The structured training and setup strategies outlined in this study may guide other teams adopting robotic platforms such as Symani® into their microsurgical practice.

## Supplementary Information

Below is the link to the electronic supplementary material.

Supplementary file1 (DOCX 17 KB)

Supplementary file2 (DOCX 25 KB)

## Data Availability

No datasets were generated or analysed during the current study.
