# Supplementary material for: Use of the Symani® microscopic surgical robot in hand surgery and operating room setup
Source: J Robot Surg. 2025 Aug 27;19(1):519. doi: 10.1007/s11701-025-02629-2 (PMC12391231; doi:10.1007/s11701-025-02629-2)
Supplement: Supplementary file 1 — Supplementary file1 (DOCX 17 KB) [file 11701_2025_2629_MOESM1_ESM.docx]

**Table 1.** Results from the System Usability Scale (SUS)

|  | Surgeon 1 | Surgeon 2 | Nurse 1 | Nurse 2 |
| --- | --- | --- | --- | --- |
| SUS1: I think that I would like to use this system frequently. | 5 | 5 | 3 | 4 |
| SUS2: I found the system unnecessarily complex. | 2 | 1 | 1 | 2 |
| SUS3: I thought the system was easy to use. | 4 | 4 | 4 | 5 |
| SUS4: I think that I would need the support of a technical  person to be able to use this system. | 4 | 5 | 5 | 5 |
| SUS5: I found the various functions in this system were well integrated | 5 | 5 | 4 | 4 |
| SUS6: I thought there was too much inconsistency in this  system. | 1 | 1 | 2 | 1 |
| SUS7: I would imagine that most people would learn to use  this system very quickly. | 4 | 4 | 4 | 3 |
| SUS8: I found the system very cumbersome to use. | 3 | 3 | 4 | 4 |
| SUS9: I felt very confident using the system. | 5 | 4 | 4 | 5 |
| SUS10: I needed to learn a lot of things before I could get  going with this system. | 2 | 3 | 1 | 2 |
| SUS score on 0 to 100 normalized scale | 77.5 | 72.5 | 65 | 67.5 |
