# Supplementary material for: Use of the Symani® microscopic surgical robot in hand surgery and operating room setup
Source: J Robot Surg. 2025 Aug 27;19(1):519. doi: 10.1007/s11701-025-02629-2 (PMC12391231; doi:10.1007/s11701-025-02629-2)
Supplement: Supplementary file 2 — Supplementary file2 (DOCX 25 KB) [file 11701_2025_2629_MOESM2_ESM.docx]

**Robotic System Checklist**

**-**

**Supplemental material for “Use of the Symani® Microscopic Surgical Robot in Hand Surgery and Operating Room Setup”**

Article title: “Use of the Symani® Microscopic Surgical Robot in Hand Surgery and Operating Room Setup”

Journal name: Journal of Robotic Surgery

Authors: Piperno, Alessandro* MD, Palombo, Enrico^+^ MD, Pagnotta, Alessia* MD, PhD

Affiliations:

*Hand and Microsurgery Unit, Jewish Hospital of Rome

^†^Department of Anatomical, Histological, Forensic Medicine and Orthopaedic Sciences, Sapienza University of Rome, Rome, Italy

Corresponding author: Alessandro Piperno, alessandropiperno@gmail.com

| **Phase 1 – Initial setup** | | ***✔*** |
| --- | --- | --- |
| 1. | Connect cables |  |
| 2. | Start up the system |  |
| 3. | Move the macropositioner from the stowed position |  |
| 4. | Center the robotic arms and electric motors (no instruments attached) |  |
| **Phase 2 - Surgical Preparation** | |  |
| 5. | Drape the robot and Symani® chair |  |
| 6. | Check NanoWrist instruments:   - Ensure tip caps are closed and correctly oriented - Remove backend caps - Inspect instrument pins |  |
| 7. | Slide instruments into the instrument connectors |  |
| 8. | Engage instruments with tip caps on (press the **Engage** button) |  |
| 9. | Position the microscope (northeast of the lead surgeon) |  |
| 10. | Position the Symani® robot (southeast of the lead surgeon), place the NanoWrist instruments near the target anatomy, and hand them over to the surgeon |  |
| 11. | Remove tip caps and verify tips are fully closed |  |
| **Phase 3 - Operation** | |  |
| 12. | Lock the wheels |  |
| 13. | Begin teleoperation |  |
| **Phase 4 - Post-Operative Procedures** | |  |
| 14. | Remove the micromanipulators from the operating field |  |
| 15. | Disengage instruments (press the **Disengage** button) |  |
| 16. | Remove instruments |  |
| 17. | Remove drapes |  |
| 18. | Return the system to the stowed position |  |
| 19. | Shut down the system |  |
| 20. | Disconnect cables |  |

**Nursing checklist**

**Robotic System Checklist**

**Surgical Checklist for Hand Procedures**

|  | | | ***✔*** |
| --- | --- | --- | --- |
| 1. | Isolation of vascular pedicles and nerves | |  |
| 2. | Microscope positioning | |  |
| 3. | Dissection of pedicles | |  |
| 4. | Symani® positioning | |  |
| 4.1 | | Sit on the draped Symani® chair and adjust height and elbow positions |  |
| 4.2 | | Position the Symani® southwest of the surgeon |  |
| 4.3 | | Place the Symani® arms close to the surgical field and position the head between them |  |
| 4.4 | | Align the microscope in front of the surgeon’s head |  |
| 4.5 | | Place the Symani® pedals under the right foot |  |
| 4.6 | | Position the microscope foot pedal (if present) under the left foot |  |
| 4.7 | | Take the Symani® pliers |  |
| 4.8 | | Establish the “connection” by pressing the blue pedal and rotating the pliers until the continuous sound stops |  |
| 5. | Venous anastomosis (if dorsal) | |  |
| 6. | Arterial and venous anastomosis/nerve sutures | |  |
| 7. | Check anastomosis patency | |  |
| 8. | Remove clamps | |  |

**TROUBLESHOOTING:**

- **Error Signal & Pliers Block** (if the surgeon moves outside of the “Symani field,” rotates the instruments excessively, and/or moves too quickly): Repeat Step 4.8.
- **Incorrect or Uncomfortable Wrist Position During Symani Use:** Press the yellow pedal (which temporary freezes the Symani instruments), adjust wrists to a comfortable position, then release the yellow pedal.
